# Long-term Outcomes and Late Effects of Definitive Chemoradiotherapy in Patients with Cervical Cancer in Nova Scotia

**DOI:** 10.7759/cureus.343

**Published:** 2015-10-06

**Authors:** Benjamin Royal-Preyra, David Bowes, Gaurav Bahl, Paul Joseph, Maureen Nolan, Hylkije Ymeri, James Bentley, Nikhilesh Patil

**Affiliations:** 1 Medical student, Dalhousie University; 2 Department of Radiation Oncology, Dalhousie University; 3 Division of Radiation Oncology and Developmental Radiotherapeutics, University of British Columbia; 4 Division of Gynecology Oncology, Dalhousie University; 5 Nova Scotia Cancer Centre, Nova Scotia Health Authority, Dalhousie University

**Keywords:** cervical cancer, brachytherapy, chemotherapy, toxicity

## Abstract

Purpose: To determine the long-term oncologic outcomes and toxicity of patients treated with definitive chemo-radiotherapy for cervical cancer.

Methods and Materials: The study period was January 1, 2000 to December 31, 2009. All patients diagnosed with cervical cancer who received curative-intent chemoradiotherapy were included. Patients were excluded if they resided out of the province, received surgery as an initial treatment, or were treated with palliative intent. A retrospective chart review was performed.

Results: Four hundred and eighty-six patients were diagnosed with cervical cancer; 190 met eligibility criteria. Median follow-up for all patients was 3.2 years (interquartile range 1.1—5.6 years). Clinical stage was FIGO IIB or higher in 139 of 190 patients (73.2%). One hundred and fifty-eight (82.7%) received concurrent cisplatin chemotherapy (mean # cycles = 4.8). The most common external beam radiotherapy (EBRT) dose/fractionation schedule was 45 Gray (Gy) in 25 fractions (149 pts, 78.0%). One hundred and thirty-six (71.2%) received low-dose-rate (LDR) brachytherapy (BT: most common dose = 35 Gy). High-dose-rate (HDR) BT was implemented in 2008; the most common HDR dose was 24 Gy in 8 fractions over five days.

Five-year overall survival (OS) and progression-free survival (PFS) were 69.4% and 61.4%, respectively. OS and PFS were significantly higher in patients who received chemotherapy vs. radiotherapy alone. For those receiving HDR-BT, there was a significantly higher OS, but not PFS. The rate of late RTOG Grade 3/4 toxicity at five years was 23.3% (gastrointestinal - 26 events, 13% of patients; genitourinary - 13 events, 8% of patients). Fourteen patients had Grade 3 radiation proctitis as the only late toxicity. EBRT dose above 45 Gy was the only factor associated with late toxicity on multivariate analysis.

Conclusion: Outcomes of patients treated with chemoradiotherapy for cervical cancer are in keeping with those reported in other series. Chemotherapy improved OS and PFS. External beam radiotherapy dose above 45 Gy was the only predictor of late toxicity.

## Introduction

Cervical cancer is a common cause of death for young women in Canada [1]. Though incidence and mortality are decreasing, largely due to screening, it remains a serious public health problem [2]. 

External beam radiotherapy and brachytherapy have long been considered the standard of care for patients with locally advanced cervical cancer. In the last 20 years, there have been two major changes in the care of patients receiving radiotherapy for cervical cancer with curative intent: the introduction of concurrent chemotherapy and the transition from low-dose-rate (LDR) to high-dose-rate (HDR) brachytherapy. Several randomized controlled trials in the 1990s demonstrated improvements in overall survival with the addition of concurrent cisplatin chemotherapy to standard radiotherapy [3]. This resulted in a National Cancer Institute (NCI) alert in 1999, which recommended widespread adoption of concurrent chemoradiotherapy [4]. Many radiotherapy centres worldwide adopted HDR brachytherapy during this time period, and HDR is now widely used in Canada [5].

The care of patients with cervical cancer in our province is coordinated by centralized Gynecologic Oncology and Radiation Oncology services. Patients with suspected or confirmed cervical cancer are seen by a gynecologic oncologist, with access to joint evaluation with radiation oncology as necessary. Patients receive external beam radiotherapy (EBRT) at one of two centres, Halifax or Sydney, which are part of the same administrative department. All patients receive brachytherapy in Halifax (which also provides brachytherapy service to the surrounding provinces on a case-by-case basis). This structure has facilitated consistent and standardized care in this patient population.

The purpose of this study was to evaluate the long-term oncologic outcomes and toxicity of patients treated with definitive radiotherapy for cervical cancer in Nova Scotia over the period between 2000 and 2009. We also sought to assess the impact of concurrent chemotherapy and HDR brachytherapy implementation.

## Materials and methods

The Capital District Health Authority ethical review board approved this study (approval #CDHA-RS/2011-237). Informed patient consents were signed at the time of treatment.

Patients were included if they were diagnosed with cervical cancer between January 1, 2000, and December 31, 2009. Patients were identified by the provincial cancer registry, which collects information on all patients diagnosed with cancer in the province of Nova Scotia. Patients were excluded if their primary residence was outside of Nova Scotia; if surgery (hysterectomy or trachelectomy) was performed as the initial treatment; if brachytherapy was not delivered; and if treatment was delivered with palliative intent (as determined on chart review by D.B.).

A retrospective chart review was undertaken for all identified patients, collecting disease variables (stage, tumor size, histology) as well as the details of treatment. Demographic information was collected from the provincial cancer registry. Survival information was obtained from the provincial department of vital statistics (censored after December 31, 2013).

Up until mid-2009, paper charts existed which contained all of the original treatment records (including radiotherapy plans and chemotherapy orders). After mid-2009, this information was collected on an electronic medical record. The electronic records were fully available for those treated at the Halifax site, and partly available for those treated in Sydney (which represents _~_1/5 of the provincial population). All brachytherapy was delivered in Halifax, and complete records documenting this component of therapy were available throughout the study period.

All cases of toxicity were reviewed by a gynecologic radiation oncologist (D.B. or G.B.) using RTOG scales [6]. This included rectal bleeding requiring intervention. Patients were recorded as having toxicity if they met the definition for either Grade 3 or 4. The individual grade (3 or 4) was not recorded. Genitourinary (GU) and gastrointestinal (GI) events were recorded and analyzed separately. Vaginal toxicity was not recorded.

All patients with a diagnosis of cervical cancer are initially seen by gynecologic oncology in a shared clinic with radiation oncology. Patients receiving primary radiotherapy are assessed by gynecologic oncology in Halifax or medical oncology in Sydney for potential concurrent chemotherapy. Staging imaging, sigmoidoscopy, and cystoscopy were not routinely performed. The assigned clinical stage was determined from the consultation notes at the time of physical examination. 

EBRT was delivered using a four-field box using 18 MV photons in all patients. No midline block was used in any patients. Patients receiving LDR brachytherapy underwent a single implant under general anaethesia, with treatment being delivered in a shielded inpatient room. HDR brachytherapy was implemented in 2007; patients receiving HDR had applicators placed once under general anaesthesia, with fractionated treatment being delivered over the course of a week. Two-dimensional planning was done using plain x-ray imaging for all patients, with the prescription dose being applied at point A. All patients underwent brachytherapy after EBRT and chemotherapy were completed. Patients were routinely seen in clinic approximately six weeks after completion and every three months for the first two years. After two years, follow-up was extended to every six months to one year depending on the patient's clinical condition.

Overall survival (OS) and progression-free survival (PFS) were calculated from the last day of therapy to the time of documented recurrence or death. Recurrence was determined based on the clinical notes, imaging records, and pathology if applicable. SPSS version 21 was used for all statistical analysis. Survival and toxicity curves were generated using Kaplan-Meier method, and Cox regression analysis was done to analyze the effect of patient and treatment factors on survival. P values < 0.05 were considered statistically significant. Factors considered in multivariable analysis for survival were stage (Stages I and IIA vs. 2B and higher), HDR vs. LDR brachytherapy, use of chemotherapy, EBRT dose (45 Gy and lower vs. above 45 Gy), location of EBRT delivery, and histology. Factors considered for toxicity evaluation were stage (Stages I and IIA vs. 2B and higher), HDR vs. LDR brachytherapy, use of chemotherapy, and applicator type.

## Results

Four hundred and eighty-six patients were diagnosed with cervical cancer during the study period. One hundred and ninety met the eligibility criteria. The median follow-up for all patients was 3.2 years (interquartile range 1.1 – 5.6 years). For patients who were alive as of December 31, 2013, the median follow-up was 4.5 years (IQ range 3.1 – 7.0 years), and the mean follow-up was 5.1 years. Information on patient demographics, histology, and tumor stage is shown in Table 1.

**Table 1:** Patient and disease characteristics

Mean Age (Range)	52 years (22-87 years)	
Histology	Squamous cell	145 (76%)
	Adenocarcinoma	28 (15%)
	Adenosquamous	11 (6%)
	Other	6 (3%)
Stage	Unknown	2 (1%)
	1A	0
	1B1	13 (7%)
	1B2	27 (14%)
	2A	9 (5%)
	2B	90 (47%)
	3A	3 (2%)
	3B	44 (23%)
	4A	1 (0.5%)
	4B	1 (0.5%)

The details of EBRT and brachytherapy fractionation schedules are shown in Table 2. The mean overall treatment time (from the start of treatment to completion) was 51 days, the median was 49 days, and interquartile range: 44-52 days. One hundred and twenty-seven patients (81%) received concurrent cisplatin chemotherapy; the mean number of cycles delivered was 4.8. One hundred and thirty-nine patients (73%) of patients received EBRT at the Halifax site; the rest received EBRT in Sydney.

**Table 2:** EBRT and brachytherapy doses and applicators used * Other EBRT schedules included 50 Gy in 25 fractions, 50-51 Gy in 28 fractions, 55.8 Gy in 31 fractions, and 40 Gy in 15 fractions. ** Other HDR brachytherapy schedules included 20 Gy in 5 fractions over 5 days, 21 Gy in 7 fractions over 5 days, 20 Gy in 4-5 fractions over 4-5 days, 20 Gy in 8 fractions over 5 days, and 25 Gy in 5 fractions over 5 days. EBRT = external beam radiotherapy LDR = low-dose rate brachytherapy HDR = high-dose rate brachytherapy Gy = Gray pts = patients

EBRT Dose	45 Gy in 25 Fractions	149 Patients (78%)
	50.4 Gy in 28 fractions	31 (16%)
	Other*	10 (5%)
Brachytherapy type	LDR	136 (71%)
	HDR	54 (28%)
LDR dose	35 Gy	105 (77% of LDR pts)
	40 Gy	25 (18% of LDR pts)
HDR dose	24 Gy in 8 fractions (over 5 days)	40 (74% of HDR pts)
	Other**	14 (26% HDR pts)
Brachytherapy applicator used	Tandem and cylinder	141 (74%)
	Tandem and ring	36 (19%)
	Other or unknown	13 (7%)

**Figure 1:**
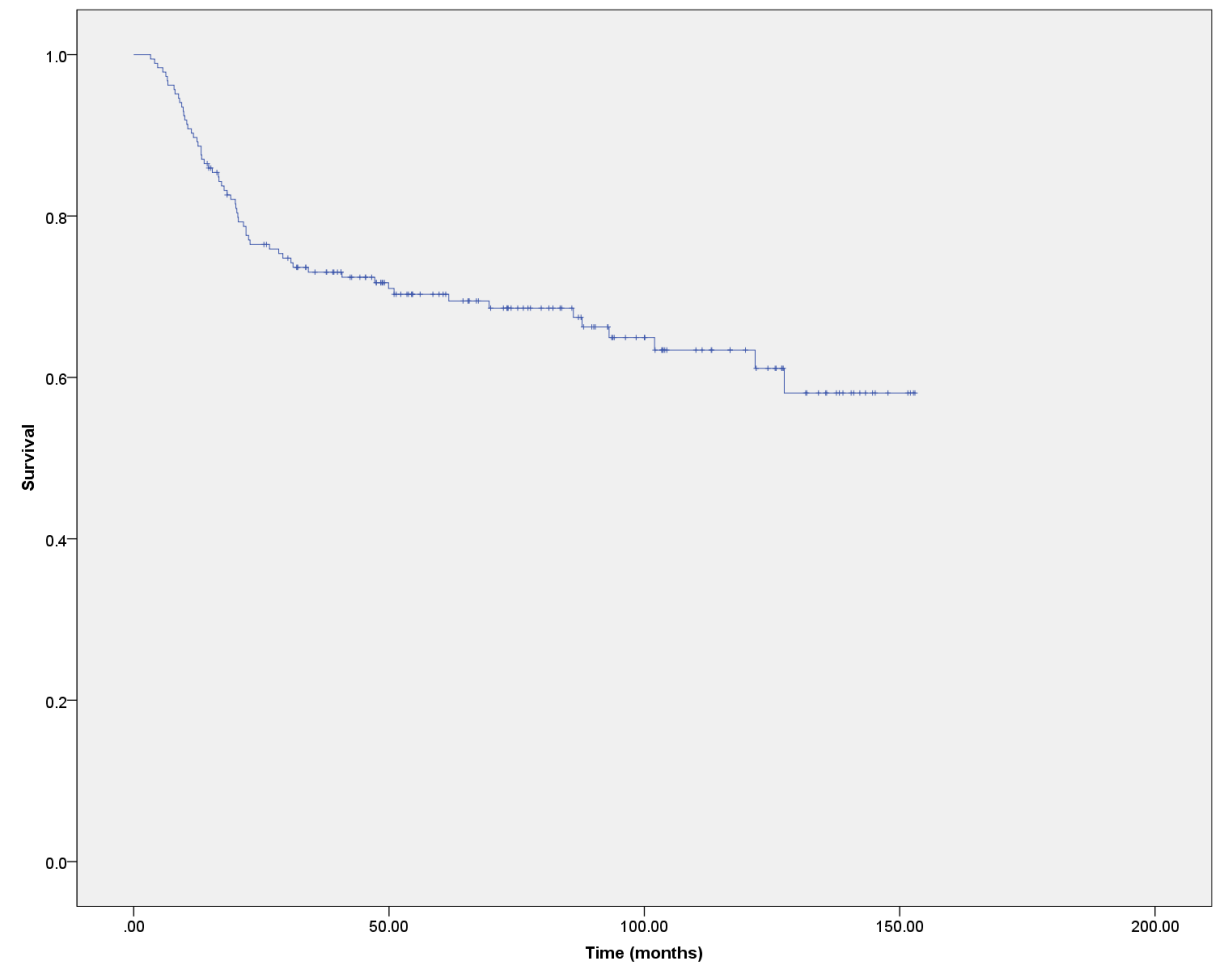
Overall survival for the entire cohort

**Figure 2:**
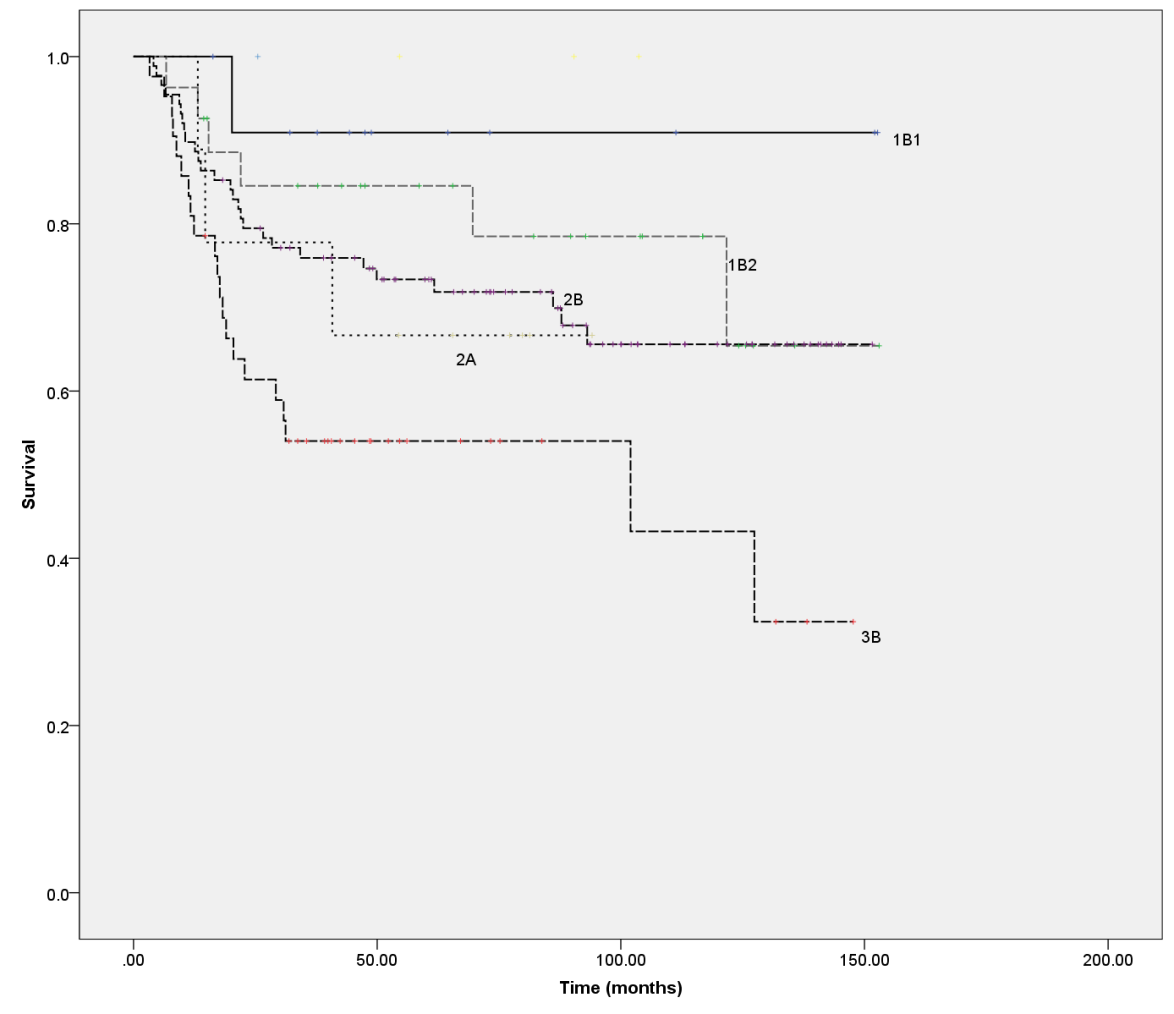
Overall survival by stage The number of patients diagnosed with each stage of disease is listed in Table 1. There were no patients with Stage 1A disease, and the numbers of patients having Stage 3A, 4A, and 4B or an unknown stage were small (3 or less)

**Figure 3:**
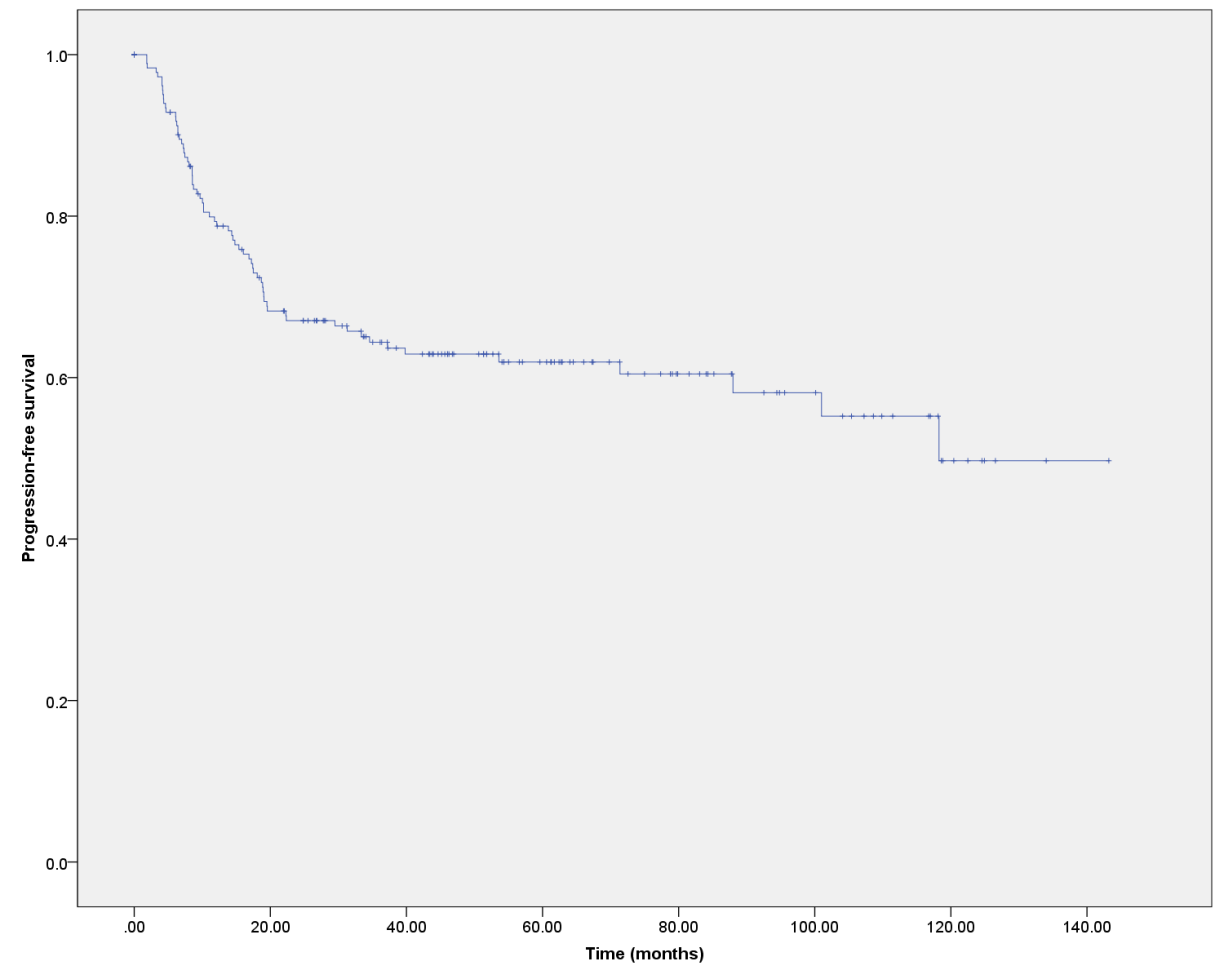
Progression-free survival (PFS) for the entire cohort

The overall survival for the cohort as a whole was 73.0% at three years and 69.5% at five years (Figure 1). The OS by disease stage is shown in Figure 2. Progression-free survival was 64.4% at three years and 61.9% at five years (Figure 3).

For patients receiving chemotherapy, the OS was noted to be 78.1% at three years and 73.4% at five years compared with 66.6% at three years and 46.4% at five years in those who did not receive chemotherapy. This met statistical significance (p = 0.047). The PFS was noted to be 81.0% at three years and 70.7% at five years in those receiving chemotherapy compared with 41.6% at three years and five years in patients who did not receive concurrent chemotherapy. This also met statistical significance (p = 0.031).

Overall survival for patients receiving HDR brachytherapy was 82.9% at three years compared with 74.6% for patients receiving LDR (p = 0.028). Progression-free survival at three years was 72.5% for patients receiving HDR and 78.9% for patients receiving LDR (p = not significant). There were no patients receiving HDR who had five years of follow-up data at the time of analysis

No factors were found to be associated with improved OS on multivariable analysis, including chemotherapy use, HDR brachytherapy (vs. LDR), stage, EBRT dose, location of EBRT delivery (Halifax or Sydney), and histology. On multivariable analysis of PFS, the only factor associated with a worse outcome was disease Stage 2B or higher.

**Figure 4:**
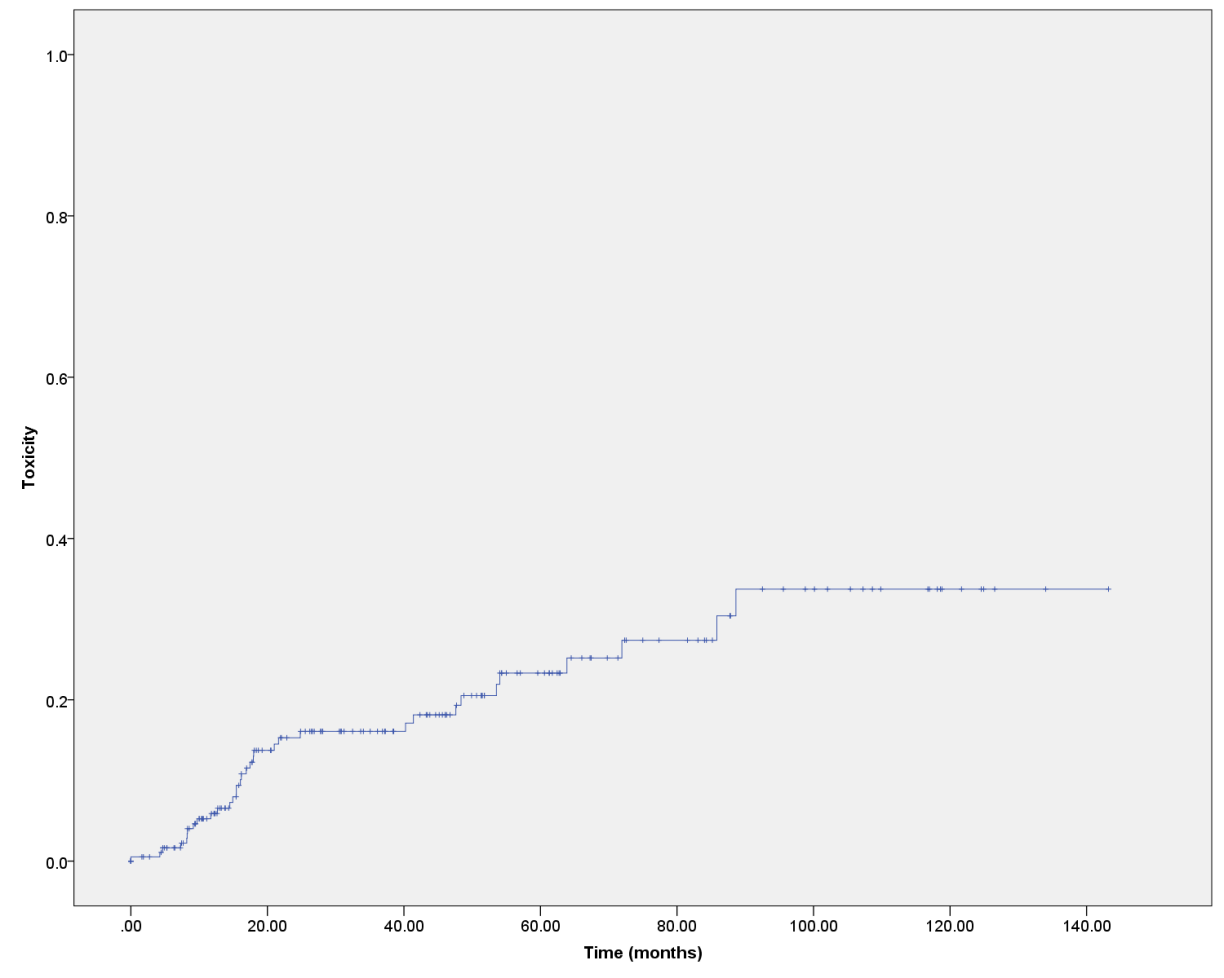
Grade 3 or 4 toxicity Rate of development of any GU or GI Grade 3 or 4 toxicity event. Multiple events in one patient are not recorded separately in this graph. This is the worst toxicity score recorded at any time in follow-up. Vaginal toxicity was not included.

The likelihood of experiencing any late Grade 3 or 4 toxicity event was 16.1% at three years post-treatment and 23.3% at five years (Figure 4). GU toxicity was noted in 5.2% of patients by three years and 7.8% by five years. GI toxicity was noted in 9.6% by three years and 12.5% by five years. Due to incomplete records, it was not possible to determine whether the toxicities resolved for most patients who experienced toxicity. In nine of 23 patients who experienced GI toxicity, the event was recorded as radiation proctitis, which either resolved after endoscopic therapies or where follow-up after endoscopic therapy was not available to determine response or recurrence. On multivariable analysis, the only factor which was associated with toxicity was receipt of EBRT dose over 45 Gy.

## Discussion

This study reports the incidence, outcomes, and late effects in patients treated for cervical cancer with external beam radiotherapy and brachytherapy in a Canadian province. In this group of patients treated over a 10-year period, the overall survival at three and five years post-therapy was 73.0% and 69.5%. Progression-free survival was 64.4% at three years and 61.9% at five years. These long-term outcomes are in keeping with those reported in other Canadian centres[7-8].

This study was limited in its retrospective nature. We were able to access original treatment records for all patients, although in many cases there was not enough information available to properly document toxicity. We were not able to accurately assess local control based on the available information. No routine imaging procedure was followed in staging or in follow-up, which may reflect both access issues during the period of the study and the absence of universal guidelines defining the role for these studies in diagnosis and follow-up after radiotherapy. The presence of recurrence, any second line or palliative therapies, and survival data are felt to be robust due to linkages with population-based databases. 

These population-based results demonstrate that chemotherapy use was widely adopted after the National Cancer Institute alert in 1999. Over 80% of patients in this study received chemotherapy, and most patients for whom data was available received all five planned cycles. Similarly, high uptake of chemotherapy was observed in Ontario, where chemotherapy use was associated with improved overall survival at a population level [9]. Our province has a centralized gynecologic oncology service with medical oncologists supervising care at the Sydney site; the centralized nature of care may explain high rates of chemotherapy receipt. Reasons for not receiving chemotherapy were not documented. Improvements in outcomes were seen on univariate analysis, which is in support of the findings observed in the randomized trials [3].

HDR brachytherapy was implemented at our centre in 2007. The impact of HDR implementation has been well documented, and the literature suggests HDR to be equivalent to LDR with respect to cancer control and toxicity [10]. In our series, patients who received HDR were noted to have a higher overall survival. This finding was unexpected, but could be explained by the fact that the number of patients treated with HDR was relatively small as well as the fact that this was a sequentially treated cohort. Our results may not fully reflect what would be observed with larger patient numbers or with longer follow-up, as we may simply be capturing other differences in the medical care of patients treated later during this time period. Our observation is reassuring in that there is no suggestion of a negative impact of HDR on disease control or toxicity. It is worth noting that several HDR dose-fractionation schedules were used in our series. Because small patient numbers were treated with each, it was not possible to perform a meaningful analysis comparing the different fractionation schedules which were used. HDR was implemented during a time when there were questions about the resource capacity for multiple fractions of therapy requiring general anaesthesia in the same patient, and these schedules were chosen to try and provide treatment which was similar to the LDR experience. This is no longer our practice, as will be discussed below.

The rate of toxicity observed in this study is significant, with 23.3% of patients available for follow-up having experienced a late Grade 3 or 4 toxicity event at five years post-treatment. It is unclear what impact the incompleteness of medical records has had on this finding. For many cases, we had difficulty assigning a proper toxicity grade retrospectively. It was particularly challenging to make the distinction between Grade 3 and 4 toxicities, so a single assignment of Grade 3/4 toxicity was made. It was also difficult to determine whether the toxicity had resolved. It was noted that for many patients, management of toxicities took place outside of the tertiary care centre where medical records were available. This raises the question as to whether we may actually be underestimating the toxicity rate. Another potential confounder is that patients who experience toxicity may be more likely to be maintained in long-term follow-up (and thereby have their toxicity events recorded). The utility of the RTOG toxicity grading system has been questioned by others, and future efforts should include measurements of patient reported outcomes [11-12]. These issues highlight the importance of the prospective collection of toxicities and quality of life data.

Despite the difficulties we noted in collecting toxicity data retrospectively, there are areas for improvement that can be identified from our findings. This study has captured the outcomes in patients treated over a 10-year period in which new technologies and therapies were implemented. There have been further advances, which have been implemented since 2010, which have the potential to lower toxicity further. The HDR brachytherapy schedule used in this population involved fractionated treatments using a single implant and two-dimensional treatment planning, which may have resulted in suboptimal repair between brachytherapy fractions and potentially high doses to organs at risk. CT-based planning has since been implemented at our centre, allowing calculation of the dose delivered to organs at risk and adjustment of treatment plans as appropriate. Other centres have demonstrated high rates of cancer control with low toxicity after incorporating MRI into treatment planning, and this may be considered at our institution in the future [13-14]. Noting that use of a tandem and cylinder applicator was commonly used in our series, the toxicity could be reduced further with more frequent use of a tandem and ring/ovoid application when possible, which would allow reduction of the biologically equivalent dose to the rectum. Use of a standardized schedule with multiple implants would also be more convenient for the patient and would be expected to lower toxicity by allowing repair of sublethal damage between fractions. Many schedules are used in clinical practice [15]. At present, our typical schedule is to deliver 45 Gy of EBRT (at 1.8 Gy per fraction, 5 fractions per week) with four HDR brachytherapy fractions of 6-7 Gy each, with the HDR and EBRT being interspersed to achieve overall treatment time under eight weeks.

Use of EBRT doses over 45 Gy was the only factor predictive of long-term toxicity on multivariate analysis. This finding has been observed in other series [16-17]. The dilemma is that any dose above 45 Gy puts the patient at risk for receiving doses to the bladder, rectum, and sigmoid in excess of what is commonly tolerated. The risk of GI toxicity rises substantially above equivalent doses of 70-75 Gy [18], and we commonly observe that the dose estimates for rectum and sigmoid are in this range even with standard EBRT and HDR doses (45 Gy EBRT + HDR brachytherapy 7 Gy x 4). Our current practice is now to recommend EBRT dose escalation only to patients who require boost EBRT doses to involved lymph nodes and for the very rare cases where brachytherapy is not technically possible. It is worth noting that other groups have reported that interstitial brachytherapy may be preferable to EBRT boost in many cases of locally advanced disease to adequately cover areas not covered in a typical tandem and ring/ovoid dose distribution [19].

## Conclusions

The outcomes of patients receiving radical external beam radiotherapy and brachytherapy in Nova Scotia between 2000 and 2009 are in keeping with those observed in other series. Our data supports the use of chemotherapy and demonstrates high uptake of chemotherapy implementation. No detrimental effect of HDR implementation was suggested. Improvements in brachytherapy delivery should be considered with the intention to improve outcomes and to reduce toxicity.
